# Correction: Accelerometer measured physical activity and the incidence of cardiovascular disease: Evidence from the UK Biobank cohort study

**DOI:** 10.1371/journal.pmed.1003809

**Published:** 2021-09-29

**Authors:** Rema Ramakrishnan, Aiden Doherty, Karl Smith-Byrne, Kazem Rahimi, Derrick Bennett, Mark Woodward, Rosemary Walmsley, Terence Dwyer

In subsequent data analyses associated with this article, the authors became aware of a coding error due to which there was an underadjustment for age. As such the following corrections are required.

In the Methods and findings subsection of the Abstract the fifth sentence is incorrect.

The correct sentence is: Hazard ratios (HRs) and 95% confidence intervals for increasing quarters of the PA distribution relative to the lowest fourth were for moderate-intensity PA: 0.79 (0.73, 0.86), 0.70 (0.64, 0.76), and 0.58 (0.53, 0.65); for vigorous-intensity PA: 0.79 (0.72, 0.86), 0.65 (0.59,0.72), and 0.56 (0.50,0.62); and for total volume of PA: 0.79 (0.73, 0.86), 0.72 (0.66, 0.79), and 0.60 (0.54, 0.66).

In the Results the third sentence of the second paragraph is incorrect.

The correct sentence is: Compared with the lowest category of moderate-intensity PA, the HRs and 95% Cis for increasing quarters were 0.79 (0.73, 0.86), 0.70 (0.64, 0.76), and 0.58 (0.53, 0.65) (Fig 1), and the corresponding values for vigorous activity were: 0.79 (0.72, 0.86), 0.65 (0.59,0.72), and 0.56 (0.50,0.62).

In the Subgroup, secondary, and sensitivity analyses subsection of the Results the second sentence of the fourth paragraph is incorrect.

The correct sentence is: Furthermore, sensitivity analyses conducted to examine the inclusion of additional covariates (hypertension, self-rated health, BMI, total cholesterol, HDL, LDL, triglycerides, C-reactive protein, HbA1c, red and processed meat, fresh fruit, and cooked vegetables) to the multivariable Cox models utilised in the main analyses attenuated the HRs by just 6.0% to 20.0% (S2–S4 Tables).

In the Discussion the second sentence of the first paragraph is incorrect.

The correct sentence is: We found broadly similar associations within each subcomponent of CVD, and the associations were comparable for men and women, with the exception of vigorous activity where there was a 14% lower risk for incident CVD among men compared with 27% lower risk among women for the second quarter compared to the first.

The following figures are incorrect: Figs 1, 2, 3, 4 and S2. The authors have provided corrected versions here.

**Fig 1:**
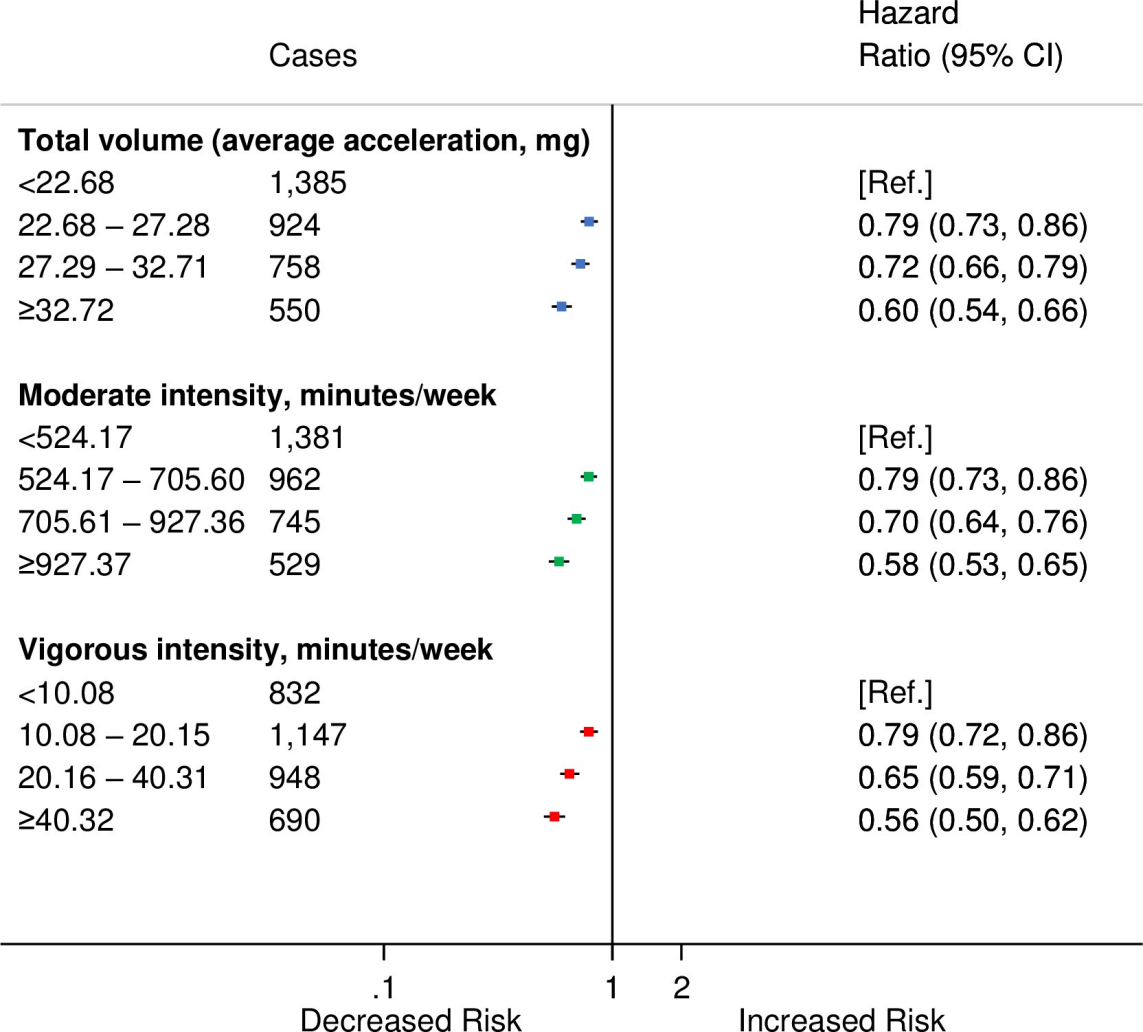
HRs^a^ for incident CVD by quarters of average accelerometer-measured total volume, moderate-intensity and vigorous-intensity physical activities in 90,211 UK Biobank participants. ^a^Adjusted for age (stratified by 5-year age-at-risk intervals), sex, ethnicity, education, Townsend Deprivation Index, smoking, and alcohol consumption. CI, confidence interval; CVD, cardiovascular disease; HR, hazard ratio. https://doi.org/10.1371/journal.pmed.1003487.g001.

**Fig 2:**
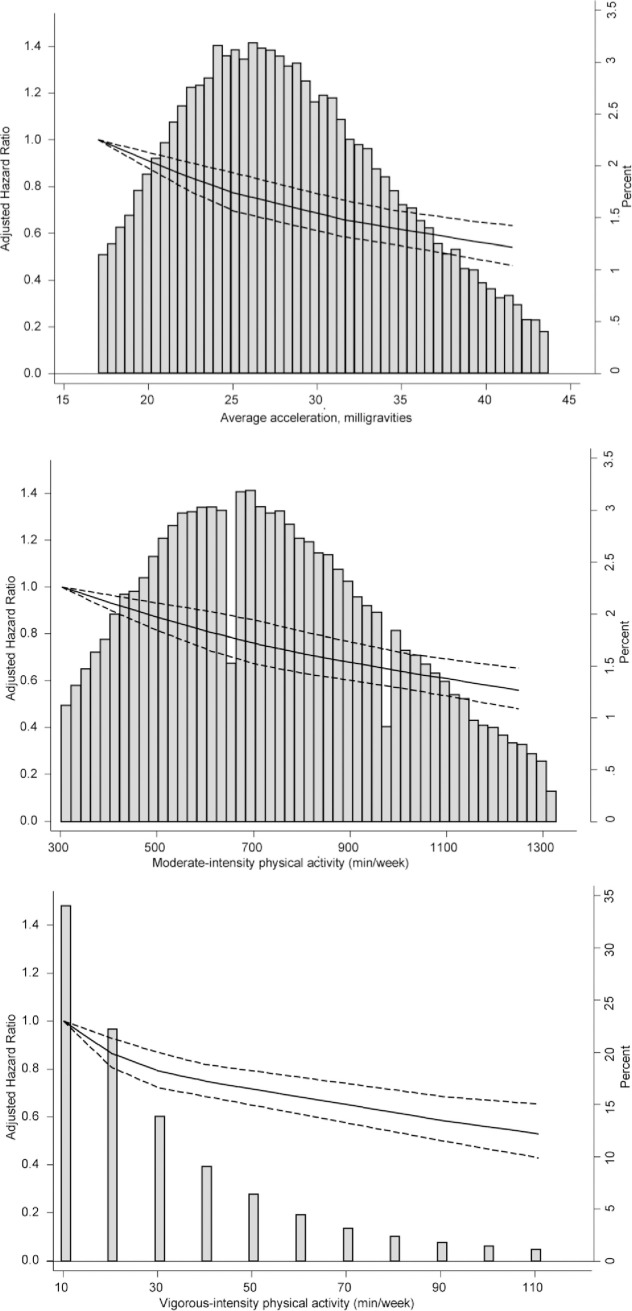
Dose–response association (HRs and associated 95% confidence interval band) between accelerometer-measured (A) total volume of PA, (B) moderate-intensity PA, and (C) vigorous-intensity PA and incident CVD using restricted cubic splines with knots at 25th, 50th, and 75th centiles of the distribution of PA (reference category = 17 milligravities (mg) for total volume of PA; 302. 4 minutes/week for moderate intensity PA; 10.08 week for vigorous intensity PA). Also shown are histograms of PA for total volume of PA in milligravities and for moderate-intensity and vigorous-intensity PA in minutes/week. CVD, cardiovascular disease; HR, hazard ratio; PA, physical activity. https://doi.org/10.1371/journal.pmed.1003487.g002.

**Fig 3:**
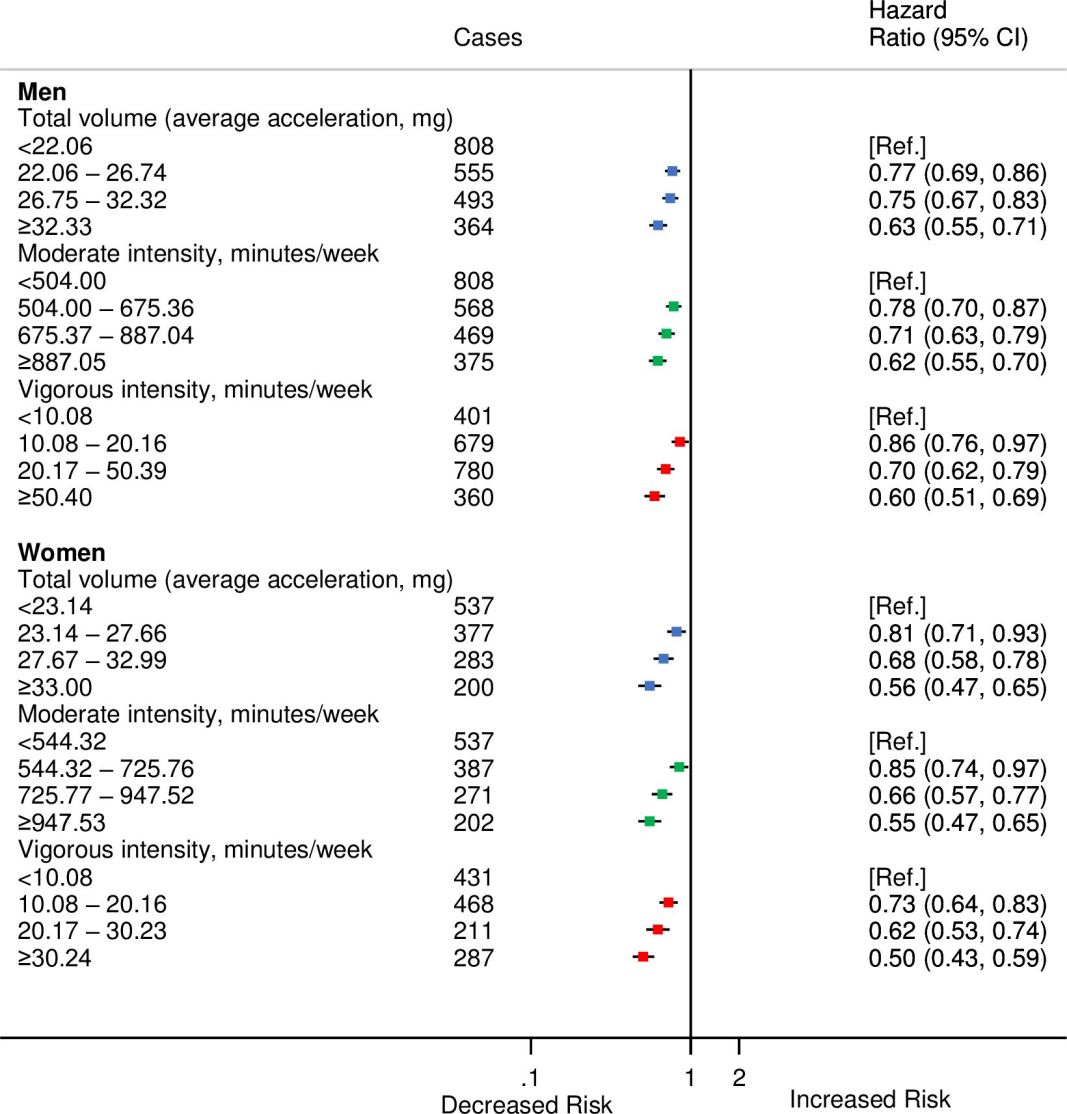
HRs^a^ for incident CVD by quarters of accelerometer-measured total volume, moderate, and vigorous physical activities stratified by sex in 90,211 UK Biobank participants. ^a^Adjusted for age (stratified by 5-year age-at-risk intervals), ethnicity, education, Townsend Deprivation Index, smoking, and alcohol consumption. https://doi.org/10.1371/journal.pmed.1003487.g003.

**Fig 4:**
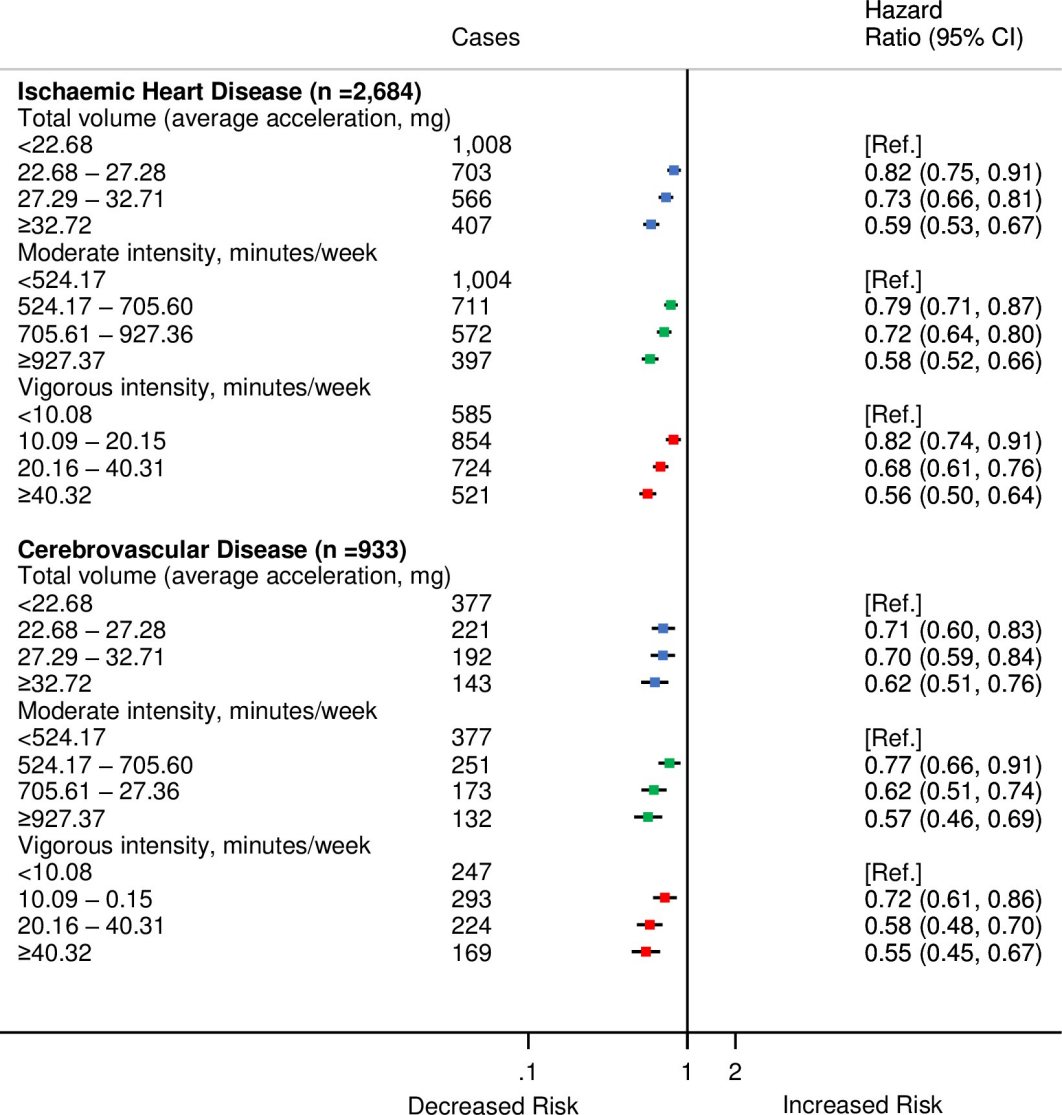
HRs^a^ for incident CVD by quarters of average accelerometer-measured total volume, moderate, and vigorous physical activities for IHD and cerebrovascular disease in 90,211 UK Biobank participants. ^a^Adjusted for age (stratified by 5-year age-at-risk intervals), sex, ethnicity, education, Townsend Deprivation Index, smoking, and alcohol consumption. CI, confidence interval; CVD, cardiovascular disease; HR, hazard ratio; IHD, ischaemic heart disease. https://doi.org/10.1371/journal.pmed.1003487.g004.

The following tables are incorrect: S1, S2, S3 and S4 Tables. The authors have provided corrected versions here.

## Supporting information

S2 Fig HRs^a^ for incident CVD by quarters of average accelerometer measured total volume, moderate, and vigorous physical activities after excluding participants who had comorbid diseases^b^ at baseline. ^a^Adjusted for age (stratified by 5-year age-at-risk intervals), sex, ethnicity, education, Townsend Deprivation Index, smoking, and alcohol consumption. ^b^Cancer (ICD codes: C01-C26, C30-C58, C60-C97, and D00-D48), diabetes mellitus (ICD codes: E10-E14), hypertension (ICD codes: I10), and chronic lower respiratory disease (ICD codes: J43 and J44.9). CVD, cardiovascular disease; HR, hazard ratio; ICD, International Classification of Diseases. https://doi.org/10.1371/journal.pmed.1003487.s007
(PDF)

S1 Table Adjusted HRs for incident CVD by quarters of average accelerometer-measured total volume (mg), moderate, and vigorous PA after removal of incident CVD occurring within 1 and 2 years of follow-up. CVD, cardiovascular disease; HR, hazard ratio; PA, physical activity.
(PDF)

S2 Table HRs for the association between quarters of total volume of PA (mg) and incident CVD with sequential adjustment for potential confounders and mediators. CVD, cardiovascular disease; HR, hazard ratio; PA, physical activity.
(PDF)

S3 Table HRs for the association between quarters of moderate PA (minutes/week) and incident CVD with sequential adjustment for potential confounders and mediators. CVD, cardiovascular disease; HR, hazard ratio; PA, physical activity.
(PDF)

S4 Table HRs for the association between quarters of vigorous PA (minutes/week) and incident CVD with sequential adjustment for potential confounders and mediators. CVD, cardiovascular disease; HR, hazard ratio; PA, physical activity.
(PDF)
