# Supplementary material for: Accelerometer measured physical activity and the incidence of cardiovascular disease: Evidence from the UK Biobank cohort study
Source: PLoS Med. 2021 Jan 12;18(1):e1003487. doi: 10.1371/journal.pmed.1003487 (PMC7802951; doi:10.1371/journal.pmed.1003487)
Supplement: S2 Table — CVD, cardiovascular disease; HR, hazard ratio; PA, physical activity. (PDF) [file pmed.1003487.s003.pdf]

S2 Table. Hazard Ratios for the association between quarters of total volume of physical activity (mg) and incident cardiovascular disease with sequential adjustment for potential confounders and mediators

**Adjustments**	**HR (95% CI)**	**HR (95% CI)**	**HR (95% CI)**
**Milli-gravity (mg)**	**22.68 – 27.28 vs ≤22.67**	**27.29-32.71 vs ≤22.67**	**>32.71 mg ≤22.67**
+ Age	0.68 (0.63, 0.74)	0.58 (0.53, 0.63)	0.44 (0.39, 0.48)
+ Sex	0.71 (0.65, 0.77)	0.61 (0.55, 0.66)	0.46 (0.41, 0.51)
+ Education	0.72 (0.65, 0.78)	0.61 (0.56, 0.67)	0.46 (0.42, 0.51)
+ Townsend Deprivation Index	0.72 (0.66, 0.78)	0.61 (0.56, 0.67)	0.46 (0.42, 0.51)
+ Ethnicity	0.72 (0.66, 0.78)	0.61 (0.56, 0.67)	0.46 (0.42, 0.51)
+ Smoking	0.72 (0.66, 0.79)	0.62 (0.57, 0.68)	0.46 (0.42, 0.51)
+ Alcohol consumption	0.73 (0.67, 0.79)	0.63 (0.57, 0.69)	0.47 (0.43, 0.52)
+ Hypertension	0.73 (0.67, 0.79)	0.63 (0.57, 0.69)	0.47 (0.43, 0.53)
+ Self rated health	0.76 (0.70, 0.83)	0.67 (0.61, 0.73)	0.51 (0.46, 0.57)
+ Body Mass Index	0.78 (0.72, 0.85)	0.69 (0.63, 0.75)	0.54 (0.49, 0.60)
+ Total cholesterol	0.78 (0.71, 0.85)	0.68 (0.62, 0.75)	0.54 (0.48, 0.60)
+ HDL cholesterol	0.78 (0.71, 0.85)	0.71 (0.64, 0.78)	0.55 (0.49, 0.62)
+ LDL cholesterol	0.78 (0.71, 0.86)	0.71 (0.64, 0.78)	0.55 (0.49, 0.62)
+ Triglycerides	0.78 (0.71, 0.86)	0.71 (0.64, 0.78)	0.55 (0.49, 0.62)
+ C-reactive protein	0.79 (0.72, 0.86)	0.72 (0.65, 0.79)	0.56 (0.50, 0.63)
+ HbA1c	0.79 (0.72, 0.87)	0.73 (0.66, 0.80)	0.57 (0.50, 0.64)
+ Red and processed meat intake	0.79 (0.72, 0.87)	0.73 (0.66, 0.80)	0.56 (0.50, 0.64)
+ Fresh fruit intake	0.79 (0.72, 0.87)	0.72 (0.65, 0.80)	0.56 (0.50, 0.63)
+ Cooked vegetable intake	0.79 (0.72, 0.87)	0.72 (0.65, 0.80)	0.56 (0.50, 0.63)

Abbreviations: HR, hazard ratio; CI, confidence interval; HbA1c, glycated haemoglobin
Note: C-reactive protein on log scale
