# Supplementary material for: Accelerometer measured physical activity and the incidence of cardiovascular disease: Evidence from the UK Biobank cohort study
Source: PLoS Med. 2021 Jan 12;18(1):e1003487. doi: 10.1371/journal.pmed.1003487 (PMC7802951; doi:10.1371/journal.pmed.1003487)
Supplement: S3 Table — CVD, cardiovascular disease; HR, hazard ratio; PA, physical activity. (PDF) [file pmed.1003487.s004.pdf]

S3 Table. Hazard Ratios for the association between quarters of moderate physical activity (minutes/week) and incident cardiovascular disease with sequential adjustment for potential confounders and mediators

**Adjustments**	**HR (95% CI)**	**HR (95% CI)**	**HR (95% CI)**
**Minutes/week**	**524.17-705.60 vs ≤524.16**	**705.61-927.36 vs ≤524.16**	**>927.36 vs ≤524.16**
+ Age	0.68 (0.62, 0.74)	0.55 (0.50, 0.60)	0.42 (0.38, 0.46)
+ Sex	0.69 (0.63, 0.75)	0.57 (0.52, 0.62)	0.45 (0.41, 0.50)
+ Education	0.69 (0.64, 0.76)	0.58 (0.53, 0.63)	0.45 (0.40, 0.50)
+ Townsend Deprivation Index	0.70 (0.64, 0.76)	0.58 (0.53, 0.63)	0.45 (0.41, 0.50)
+ Ethnicity	0.70 (0.64, 0.76)	0.58 (0.53, 0.63)	0.45 (0.41, 0.50)
+ Smoking	0.70 (0.65, 0.76)	0.58 (0.53, 0.64)	0.45 (0.41, 0.50)
+ Alcohol consumption	0.71 (0.65, 0.77)	0.59 (0.54, 0.64)	0.46 (0.41, 0.50)
+ Hypertension	0.71 (0.66, 0.77)	0.59 (0.54, 0.65)	0.46 (0.41, 0.51)
+ Self rated health	0.74 (0.68, 0.80)	0.63 (0.57, 0.69)	0.49 (0.44, 0.54)
+ Body Mass Index	0.76 (0.70, 0.82)	0.65 (0.59, 0.71)	0.52 (0.47, 0.57)
+ Total cholesterol	0.75 (0.69, 0.82)	0.66 (0.60, 0.72)	0.51 (0.46, 0.57)
+ HDL cholesterol	0.75 (0.69, 0.82)	0.67 (0.61, 0.74)	0.52 (0.46, 0.58)
+ LDL cholesterol	0.75 (0.69, 0.82)	0.67 (0.61, 0.74)	0.52 (0.46, 0.58)
+ Triglycerides	0.75 (0.69, 0.82)	0.67 (0.61, 0.74)	0.52 (0.46, 0.58)
+ C-reactive protein	0.76 (0.69, 0.83)	0.68 (0.62, 0.75)	0.53 (0.47, 0.59)
+ HbA1c	0.76 (0.69, 0.83)	0.69 (0.63, 0.77)	0.53 (0.47, 0.60)
+ Red and processed meat intake	0.76 (0.69, 0.83)	0.69 (0.63, 0.77)	0.53 (0.47, 0.60)
+ Fresh fruit intake	0.76 (0.69, 0.83)	0.69 (0.62, 0.77)	0.53 (0.47, 0.60)
+ Cooked vegetable intake	0.76 (0.69, 0.83)	0.69 (0.62, 0.77)	0.53 (0.47, 0.60)

Abbreviations: HR, hazard ratio; CI, confidence interval; HbA1c, glycated haemoglobin
Note: C-reactive protein on log scale
