# Supplementary material for: Accelerometer measured physical activity and the incidence of cardiovascular disease: Evidence from the UK Biobank cohort study
Source: PLoS Med. 2021 Jan 12;18(1):e1003487. doi: 10.1371/journal.pmed.1003487 (PMC7802951; doi:10.1371/journal.pmed.1003487)
Supplement: S4 Table — CVD, cardiovascular disease; HR, hazard ratio; PA, physical activity. (PDF) [file pmed.1003487.s005.pdf]

S4 Table. Hazard Ratios for the association between quarters of vigorous physical activity (minutes/week) and incident cardiovascular disease with sequential adjustment for potential confounders and mediators

**Adjustments**	**HR (95% CI)**	**HR (95% CI)**	**HR (95% CI)**
**Minutes/week**	**10.09-20.16 vs ≤10.08**	**20.17 -40.32 vs ≤10.08**	**>40.32 vs ≤10.08**
+ Age	0.74 (0.67, 0.81)	0.60 (0.54, 0.66)	0.48 (0.43, 0.54)
+ Sex	0.69 (0.63, 0.76)	0.53 (0.48, 0.58)	0.40 (0.36, 0.44)
+ Education	0.69 (0.63, 0.76)	0.52 (0.47, 0.57)	0.40 (0.36, 0.44)
**+ Townsend Deprivation Index**	0.69 (0.63, 0.76)	0.52 (0.47, 0.58)	0.40 (0.36, 0.44)
+ Ethnicity	0.69 (0.63, 0.76)	0.52 (0.47, 0.58)	0.40 (0.36, 0.44)
+ Smoking	0.70 (0.64, 0.76)	0.53 (0.48, 0.58)	0.40 (0.36, 0.45)
+ Alcohol consumption	0.70 (0.64, 0.77)	0.54 (0.49, 0.59)	0.41 (0.37, 0.46)
+ Hypertension	0.70 (0.64, 0.77)	0.54 (0.49, 0.59)	0.41 (0.37, 0.46)
+ Self rated health	0.73 (0.66, 0.80)	0.57 (0.52, 0.63)	0.45 (0.41, 0.50)
+ Body Mass Index	0.74 (0.68, 0.81)	0.59 (0.53, 0.65)	0.48 (0.43, 0.53)
+ Total cholesterol	0.75 (0.68, 0.82)	0.59 (0.54, 0.66)	0.48 (0.43, 0.53)
+ HDL cholesterol	0.73 (0.66, 0.81)	0.59 (0.53, 0.66)	0.49 (0.43, 0.55)
+ LDL cholesterol	0.73 (0.66, 0.81)	0.59 (0.53, 0.66)	0.48 (0.43, 0.55)
+ Triglycerides	0.73 (0.66, 0.81)	0.59 (0.53, 0.66)	0.48 (0.43, 0.54)
+ C-reactive protein	0.74 (0.67, 0.81)	0.60 (0.54, 0.67)	0.49 (0.44, 0.56)
+ HbA1c	0.74 (0.67, 0.82)	0.60 (0.54, 0.67)	0.50 (0.44, 0.56)
+ Red and processed meat intake	0.74 (0.67, 0.82)	0.60 (0.54, 0.67)	0.50 (0.44, 0.56)
+ Fresh fruit intake	0.74 (0.67, 0.82)	0.60 (0.54, 0.67)	0.50 (0.44, 0.56)
+ Cooked vegetable intake	0.74 (0.67, 0.82)	0.60 (0.54, 0.67)	0.50 (0.44, 0.56)

Abbreviations: HR, hazard ratio; CI, confidence interval; HbA1c, glycated haemoglobin
Note: C-reactive protein on log scale
