# Supplementary figures and images for: Accelerometer measured physical activity and the incidence of cardiovascular disease: Evidence from the UK Biobank cohort study
Source: PLoS Med. 2021 Jan 12;18(1):e1003487. doi: 10.1371/journal.pmed.1003487 (PMC7802951; doi:10.1371/journal.pmed.1003487)

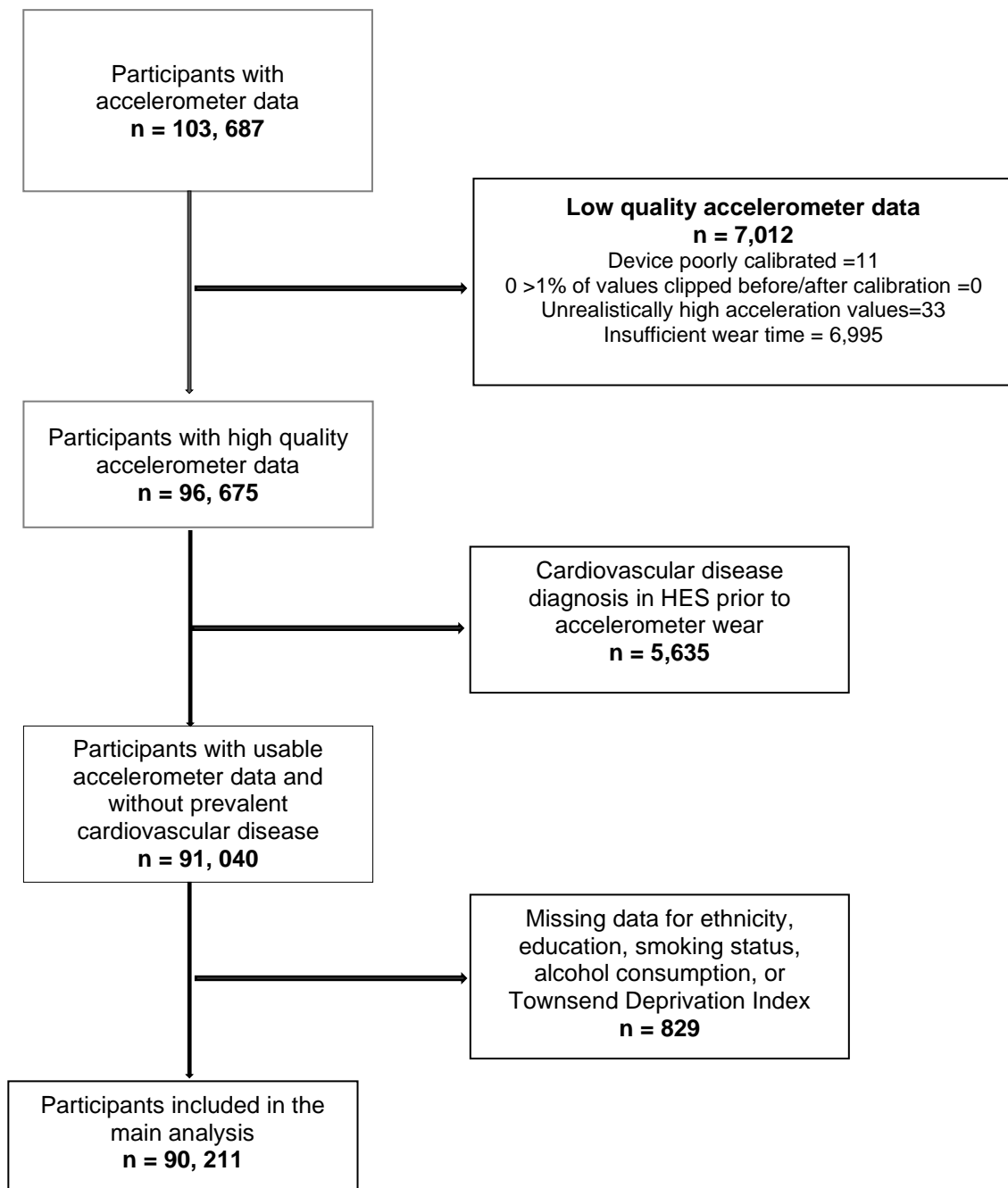

Supplement: S1 Fig — Note: The individual numbers for participant exclusion due to low quality accelerometer data do not add to 7,012 because some participants were excluded due to multiple reasons. (PDF) [file pmed.1003487.s006.pdf]

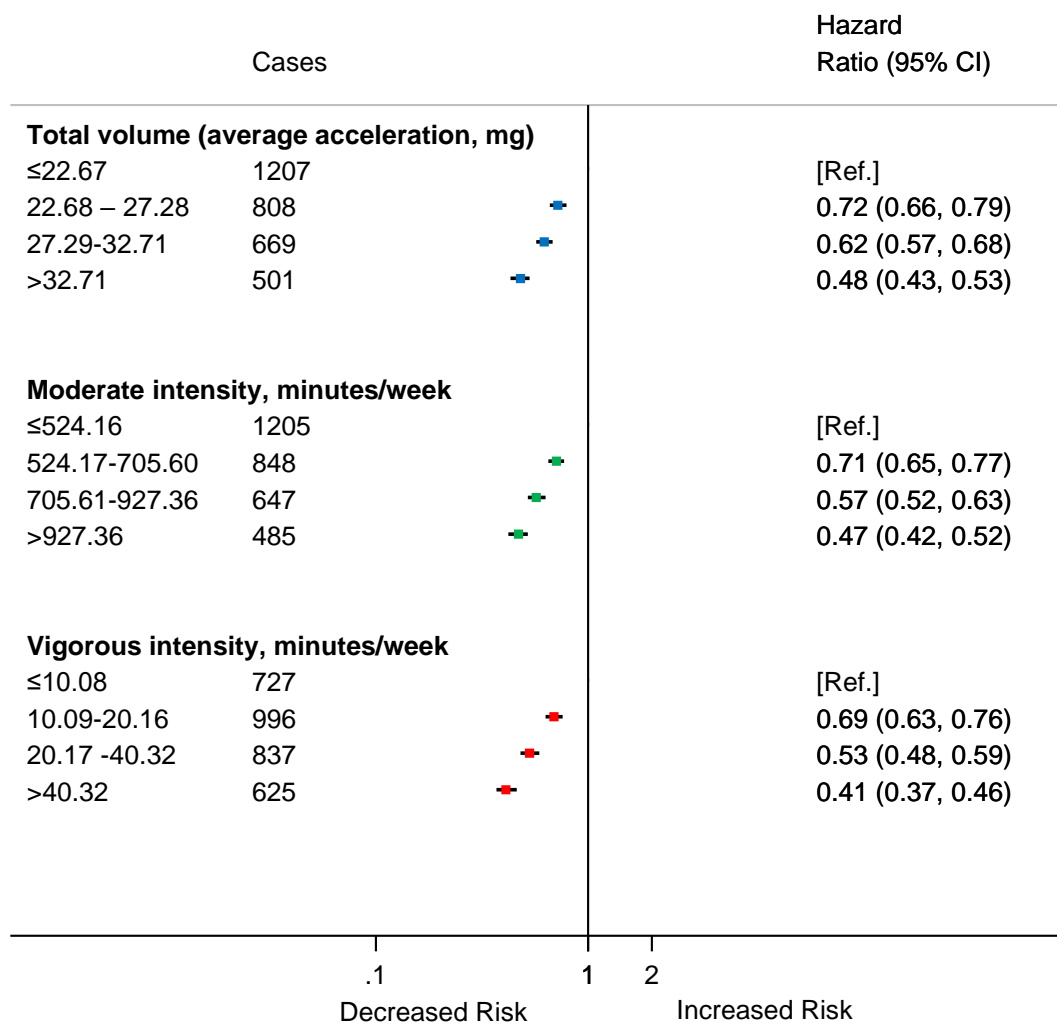

Supplement: S2 Fig — aAdjusted for age (stratified by 5-year age-at-risk intervals), sex, ethnicity, education, Townsend Deprivation Index, smoking, and alcohol consumption. bCancer (ICD codes: C01-C26, C30-C58, C60-C97, and D00-D48), diabetes mellitus (ICD codes: E10-E14), hypertension (ICD codes: I10), and chronic lower respiratory disease (ICD codes: J43 and J44.9). CVD, cardiovascular disease; HR, hazard ratio; ICD, International Classification of Diseases. (PDF) [file pmed.1003487.s007.pdf]
