# Supplementary material for: Accelerometer measured physical activity and the incidence of cardiovascular disease: Evidence from the UK Biobank cohort study
Source: PLoS Med. 2021 Jan 12;18(1):e1003487. doi: 10.1371/journal.pmed.1003487 (PMC7802951; doi:10.1371/journal.pmed.1003487)
Supplement: Attachment — Submitted filename: Response to reviewers.docx [file pmed.1003487.s008.docx]

**INCLUSION OF EXTRA FOLLOW-UP DATA**:

Just after we received the comments from the reviewers, we were notified that the UK Biobank had released updated hospital statistics and death data up to the end of May 2020. We believed that including the extra available events, from longer follow-up that more than doubled the number in the original manuscript, would further strengthen this manuscript. Therefore, we decided to re-run all the analyses using updated data.

The new dataset has 90,211 participants instead of 90,911 (the sample size in the paper we submitted) because a small number of UK Biobank participants dropped out of the study and requested that their data not be used for analysis. To add to this, the censoring date in the previous analyses was the same for all participants; however, in the current analyses the censoring dates are 31 March 2020, 29 February, 2016, and 31 October 2016 for participants from England, Wales, and Scotland, respectively. Further, in the previous version of the paper we had reported the sample size and CVD events for the entire sample whereas in the current version we have reported numbers for participants who had complete data for the exposures, outcome, and selected covariates (age, sex, ethnicity, education, Townsend Deprivation Index, smoking, and alcohol consumption).

Importantly, the extra three years of follow up has increased the number of CVD events available to 3,617 from the 1,478 in the previous analysis. These new results support the same conclusions as those reported in our original manuscript but have allowed us to estimate associations much more precisely.

**REQUESTS FROM THE EDITORS**:

**Editorial comment 1**

*At this stage, we ask that you include a short, non-technical Author Summary of your research to make findings accessible to a wide audience that includes both scientists and non-scientists. The Author Summary should immediately follow the Abstract in your revised manuscript. This text is subject to editorial change and should be distinct from the scientific abstract. Please see our author guidelines for more information: <https://journals.plos.org/plosmedicine/s/revising-your-manuscript#loc-author-summary>

**Response**

As requested, we have added a short, non-technical Author summary after the abstract.

**Editorial comment 2**

* In the last sentence of the Abstract Methods and Findings section, please briefly note any key limitation(s) of the study's methodology.

**Response**

We have added the following sentence to the end of the Methods and Findings section [Page 3, Lines 72-75]:

*We took account of potential confounders, but unmeasured confounding remains a possibility, and while removal of early deaths did not affect the estimated hazard ratios we cannot completely dismiss the likelihood that reverse causality has contributed to the findings.*

**Editorial comment 3**

*Please clarify in the Methods section if the analytical approach followed here corresponded to one laid out in a prospective protocol or analysis plan? Please state this (either way) early in the Methods section.

a) If a prospective analysis plan (from your funding proposal, IRB or other ethics committee submission, study protocol, or other planning document written before analyzing the data) was used in designing the study, please include the relevant prospectively written document with your revised manuscript as a Supporting Information file to be published alongside your study, and cite it in the Methods section. A legend for this file should be included at the end of your manuscript.

b) If no such document exists, please make sure that the Methods section transparently describes when analyses were planned, and when/why any data-driven changes to analyses took place.

c) In either case, changes in the analysis-- including those made in response to peer review comments-- should be identified as such in the Methods section of the paper, with rationale.

**Response**

Our study did not have a prespecified analysis. We have added the following text as the first sentence of the Statistical analysis section [Page 8, Line 186]:

*This study did not have a prespecified analysis plan.*

**Editorial comment 4**

*We noted the authors used the STROBE guideline to help reporting of this study, which is good - if possible, please also state this in the Methods section and cite the corresponding paper/citation (listed here - <https://www.equator-network.org/reporting-guidelines/strobe/>), and add a mention of the STROBE checklist provided as a supporting information file.

**Response**

As suggested, we have added the following sentence to the Methods section [Page 10, Lines 235-236] and have cited the paper listed above:

*This study was reported as per the Strengthening the Reporting of Observational Studies in Epidemiology (STROBE) guidelines [20] (S1 STROBE Checklist).*

**Editorial comment 5**

In addition, we request that you upload any figures associated with your paper as individual TIF or EPS files with 300dpi resolution at resubmission; please read our figure guidelines for more information on our requirements: <http://journals.plos.org/plosmedicine/s/figures>. While revising your submission, please upload your figure files to the PACE digital diagnostic tool, <https://pacev2.apexcovantage.com/>. PACE helps ensure that figures meet PLOS requirements. To use PACE, you must first register as a user. Then, login and navigate to the UPLOAD tab, where you will find detailed instructions on how to use the tool. If you encounter any issues or have any questions when using PACE, please email us at [PLOSMedicine@plos.org](mailto:PLOSMedicine@plos.org).

**Response:**

All figures included in the revised manuscript are in TIF format with 500 dpi resolution. We used the PACE digital diagnostic tool to ensure that these figures met PLOS requirements.

**Editorial comment 6**

We ask every co-author listed on the manuscript to fill in a contributing author statement, making sure to declare all competing interests. If any of the co-authors have not filled in the statement, we will remind them to do so when the paper is revised. If all statements are not completed in a timely fashion this could hold up the re-review process. If new competing interests are declared later in the revision process, this may also hold up the submission. Should there be a problem getting one of your co-authors to fill in a statement we will be in contact. YOU MUST NOT ADD OR REMOVE AUTHORS UNLESS YOU HAVE ALERTED THE EDITOR HANDLING THE MANUSCRIPT TO THE CHANGE AND THEY SPECIFICALLY HAVE AGREED TO IT. You can see our competing interests policy here: <http://journals.plos.org/plosmedicine/s/competing-interests>.

**Response:**

We have uploaded conflict of interest forms completed by each author. In addition to this, the manuscript includes a detailed contributing author statement.

**Editorial comment 7**

Please ensure that the paper adheres to the PLOS Data Availability Policy (see <http://journals.plos.org/plosmedicine/s/data-availability>), which requires that all data underlying the study's findings be provided in a repository or as Supporting Information. For data residing with a third party, authors are required to provide instructions with contact information for obtaining the data. PLOS journals do not allow statements supported by "data not shown" or "unpublished results." For such statements, authors must provide supporting data or cite public sources that include it.

**Response:**

We have now entered the following during the submission process:

*The data underlying the results presented in the study are available from the UK Biobank (*[*http://biobank.ndph.ox.ac.uk/showcase/*](http://biobank.ndph.ox.ac.uk/showcase/)

**COMMENTS FROM REVIEWER 1:**

**Comment 1**

The authors present a well-written manuscript examining the association of accelerometer-assessed physical activity and incident cardiovascular disease. Comments below.

**Response:**

Thank you.

**Comment 2**

Model analysis - age adjustment. Age is noted as stratified within the analysis but not presented by 5-year age groups. Does this mean they were calculated independently for each strata then weighted for an overall measure? Also is there an interaction by age strata compared to an inclusion of the age groupings as a standard covariate within the model?

**Response:**

We have conducted age-at-risk analyses because age-at-risk determines current risk rather than the age when the risk factor was measured or determined. As age-at-risk is a time-varying covariate it indicates that as an individual gets older during follow-up they may contribute to more than one “age-at-risk” group. All analyses were stratified by age-at-risk (in 5-year intervals) and this was done to satisfy the proportional hazards assumption within each age-at-risk group.

We have modified the text in the Methods- Statistical Analysis section to the following [Pages 8, 9, Lines 200-205]:

*Analyses were adjusted for age (stratified by 5-year age-at risk intervals to satisfy the proportional hazards assumption within each age-at-risk group), sex, ethnicity (white and non-white), age completed full time education (years), area-based social deprivation using the Townsend Deprivation Index [19] (categorized into quarters), smoking (never, former and current), and alcohol (never, less than 3 times/week, and ≥3 times/week) which were collected at baseline (2006-2010).*

**Comment 3**

Fig 2. Distribution component. It is difficult to understand these other than the relative distribution based on the minutes (horizontal axis). it is unclear why A and B are solid areas under the curve, while C is a line. Is this a histogram / cumulative density function? A secondary axis of percent of population may be informative. The presentation of the log HR is a bit atypical. is there a reason for not simply using HR, especially as Figure 1 is in this scale. If the authors prefer log HR, please indicate on the curve where the median of each quartile is so we can compare between the two figures.

**Response:**

We have revised the figure. The new figure presents the Hazard Ratio (HR) instead of the log HR and we have substituted histograms for the cumulative density function graphs**.**

**Comment 4**

Fig 3. Shouldn’t these quartiles be sex-specific? Did the authors consider using sex-specific quartiles in main analysis (fig 2)?

**Response:**

As suggested, we have computed sex-specific quartiles for the analyses stratified by sex. We have added the following text to the Methods section [Page 10, Lines 222-223]:

*For subgroup analyses by sex, we categorised total volume, and each intensity of PA into equal quarters of their distributions that were sex specific.*

**Comment 5**

Suppl Tables 2/3/4. If this is backward/forward selection of variables using a Type 1 p-value, was there an a-priori reasoning for the order of the covariates?

**Response:**

Based on literature we decided a-priori the list of variables to be included in the multivariable model as potential confounders (age, sex, ethnicity, Townsend Deprivation Index, smoking, and alcohol consumption) for the results presented in this paper. Therefore, we did not use backward/forward selection of variables. We ran consecutive models presented in Supplementary Tables 2, 3, and 4 to illustrate the effect of addition of other potential cofounders and mediators.

**Comment 6**

Dose-response. The authors compare the observed linear association to the Arem LTPA paper which observes a curvilinear association with a threshold effect at 22.5+ met/hr. So both papers show a linear response for the lower 75%. The linear vs threshold is only observed in the highest 25%. Future research may be able to better explore these differences. I am unaware of papers that have looked at the volume reported among the highest active, but the threshold could be due to differential reporting in this group compared to the lower groups.

**Response:**

We agree that the threshold for the highest 25% observed in the Arem paper, which is based on questionnaire measurement of PA, could be the result of bias associated with the way the questionnaire was answered.

**COMMENTS FROM REVIEWER 2:**

**Reviewer #2**: This is an interesting and useful study on the association between accelerometer measured physical activity and the incidence of cardiovascular disease using the UK Biobank data. The study design, datasets, statistical methods and analyses, and presentation (tables and figures) and interpretation of results are mostly adequate. However, there are still a few issues needing attention.

**Response:**

Thank you.

**Comment 1**

1) Competing risk. In the survival analyses, incident CVD was used as a primary endpoint rather than all-cause mortality. Although death from CVD was included, death from other causes became competing risk in the survival analyses. This also applies the secondary analyses for IHD and cerebrovascular disease. Can authors please show the number of deaths from non-CVD causes during the study follow up and perform competing risk analyses as appropriate?

**Response:**

We conducted competing risk analysis using cause-specific and Fine and Gray methods. The hazard ratios for cause-specific analysis were identical to the estimates that we got for models that did not account for competing risks. The hazard ratios and their associated confidence intervals for the models that used Fine and Gray method had no substantial impact on the overall results (as shown in the table below).

Therefore, we have presented the results from models that did not account for competing risks.

|  | **Cause-specific** | **Fine & Gray** |
| --- | --- | --- |
| **Overall CVD** |  |  |
| Total volume | **HR (95% CI)** | **HR (95% CI)** |
| 22.68 –27.28 vs ≤22.67 | 0.73 (0.67, 0.79) | 0.74 (0.68, 0.80) |
| 27.29-32.71 vs ≤22.67 | 0.63 (0.57, 0.68) | 0.63 (0.58, 0.69) |
| >32.71 vs ≤22.67 | 0.47 (0.43, 0.52) | 0.48 (0.43, 0.53) |
| Moderate PA |  |  |
| 524.18-705.60 vs ≤524.17 | 0.71 (0.65, 0.77) | 0.72 (0.66, 0.78) |
| 705.61-927.36 vs ≤524.17 | 0.59 (0.54, 0.65) | 0.60 (0.55, 0.65) |
| >927.4 vs ≤524.17 | 0.46 (0.41, 0.51) | 0.46 (0.42, 0.51) |
| Vigorous PA |  |  |
| 10.09-20.16 vs ≤10.08 | 0.70 (0.64, 0.77) | 0.71 (0.65, 0.78) |
| 20.17 -40.32 ≤10.08 | 0.54 (0.49, 0.59) | 0.55 (0.50, 0.60) |
| >40.32 ≤10.08 | 0.41 (0.37, 0.46) | 0.42 (0.38, 0.47) |
| **Ischaemic Heart Disease** |  |  |
| Total volume |  |  |
| 22.68 –27.28 vs ≤22.67 | 0.75 (0.68, 0.82) | 0.76 (0.69, 0.84) |
| 27.29-32.71 vs ≤22.7 | 0.63 (0.57, 0.70) | 0.64 (0.58, 0.71) |
| >32.71 vs ≤22.7 | 0.46 (0.41, 0.52) | 0.47 (0.42, 0.53) |
| Moderate PA |  |  |
| 524.18-705.60 vs ≤524.17 | 0.70 (0.64, 0.77) | 0.72 (0.65, 0.79) |
| 705.61-927.36 vs ≤524.17 | 0.60 (0.54, 0.66) | 0.62 (0.56, 0.6*) |
| >927.4 vs ≤524.17 | 0.45 (0.40, 0.50) | 0.46 (0.41, 0.52) |
| Vigorous PA |  |  |
| 10.09-20.16 vs ≤10.08 | 0.73 (0.66, 0.81) | 0.74 (0.67, 0.82) |
| 20.17 -40.32 ≤10.08 | 0.56 (0.50, 0.63) | 0.58 (0.52, 0.64) |
| >40.32 ≤10.08 | 0.41 (0.37, 0.47) | 0.42 (0.38, 0.48) |
| **Cerebrovascular Disease** |  |  |
| Total volume |  |  |
| 22.68 –27.28 vs ≤22.67 | 0.67 (0.56, 0.80) | 0.69 (0.58, 0.82) |
| 27.29-32.71 vs ≤22.7 | 0.62 (0.51, 0.74) | 0.64 (0.53, 0.77) |
| >32.71 vs ≤22.7 | 0.51 (0.42, 0.63) | 0.53 (0.43, 0.65) |
| Moderate PA |  |  |
| 524.18-705.60 vs ≤524.17 | 0.70 (0.59, 0.83) | 0.73 (0.62, 0.86) |
| 705.61-927.36 vs ≤524.17 | 0.53 (0.44, 0.64) | 0.55 (0.46, 0.67) |
| >927.4 vs ≤524.17 | 0.46 (0.37, 0.57) | 0.49 (0.39, 0.60) |
| Vigorous PA |  |  |
| 10.09-20.16 vs ≤10.08 | 0.63 (0.53, 0.75) | 0.65 (0.54, 0.77) |
| 20.17 -40.32 ≤10.08 | 0.46 (0.38, 0.5^) | 0.48 (0.39, 0.58) |
| >40.32 ≤10.08 | 0.41 (0.33, 0.51) | 0.44 (0.35, 0.54) |

We have inserted the following text in the methods section (statistical analysis) [Page 9, Lines 207-210]:

*We conducted competing risk analysis using cause-specific and Fine and Gray methods [21]. There was no material difference between the estimates from these models and the models that did not account for competing risks. Therefore, we have presented the results from the latter.*

**Comment 2**

2) Using the 7-day accelerometry measurement of activity status to represent habitual PA is a bit too simplistic. There is not much evidence apart from only one reference. The assumption here is that this 7-day activity represents a person's PA style over the years is subject to scrutiny. This is definitely a limitation and shouldn't be brushed away. Need a bit more comprehensive and critical discussion on this and its potential impact on the results.

**Response:**

We agree that there is currently a lack of robust evidence that a 7-day measurement is representative of a person’s PA for that generic period in their life. A recent paper was published that used data from 914 adults who wore a hip-worn ActiGraph GT3X (AG) twice, 6 months apart [1]. This paper found an intra-class correlation coefficient of 0.58 (0.52-0.63) for moderate-to-vigorous PA. This indicates that accelerometer-based classifications of activity are moderately stable over time, but there is considerable within-subject variability. The likely effect of this is that our reported hazard ratios are attenuated and likely to underestimate the true association between physical activity and CVD. We have now updated the discussion section as follows [Page 17, Lines 368-374]:

*There is currently a lack of robust evidence on whether a 7-day measurement is representative of habitual PA. A previous validation study showed a 7-day measure is strongly associated with PA over a period of up to 3.7 years [9]. In contrast, a recent study found that moderate-to-vigorous PA measured by hip-worn accelerometers is moderately stable over time, but there is considerable within-subject variability [32]. It is therefore possible that if the measurement error is random, our findings are attenuated and have underestimated the true association between physical activity and CVD.*

[32] Saint-Maurice PF, Sampson JN, Keadle SK, Willis EA, Troiano RP, Matthews CE. Reproducibility of Accelerometer and Posture-derived Measures of Physical Activity. Med Sci Sports Exerc. 2020 Apr;52(4):876–83.

**Comment 3**

3) Throughout the paper, in a few figure legends, it says "Stratified by age-at-risk and adjusted for ethnicity, education, Townsend Deprivation Index, smoking, and alcohol consumption", which seems a bit awkward. Should it simply be "Adjusted for age, ethnicity, education,..."?

**Response:**

As given above for response two to reviewer #1, all analyses were stratified by age-at-risk (in 5-year intervals) to satisfy the proportional hazards assumption within each age-at-risk group. The age-at-risk determines current risk rather than the age when the risk factor was measured or determined. As age-at-risk is a time-varying covariate it indicates that as an individual gets older during follow-up they may contribute to more than one “age-at-risk” group. However, we have modified the figures legends to*:*

*Adjusted for age (stratified by 5-year age-at risk intervals), sex, ethnicity, education, Townsend Deprivation Index, smoking, and alcohol consumption.*

**Comment 4**

4) Also, in the sensitivity analyses, it is a bit uncomfortable to remove 10,720 participants with either cancer, diabetes mellitus, hypertension, or chronic lower respiratory. Why not just adjusted these 4 conditions in the analyses?

**Response:**

We conducted this analysis and since there was negligible difference between these results and the results after exclusion of these individuals with comorbid conditions we have retained results from the analyses that excluded these participants.

**COMMENTS FROM REVIEWER 3:**

**REVIEWER #3:** This is a well conducted epidemiologic study examined the associations of physical activity measured via accelerometry with incident cardiovascular disease. This study provides important contributions to the literature with its longitudinal design, accelerometer measured physical activity and large sample.

Other major strengths of this study include that the authors examined physical activity based on various intensities including total PA, moderate and vigorous PA, finding that all intensity had similar linear inverse associations with incident CVD. The authors also included analyses that account for many covariates covering socioeconomic status, other lifestyle behaviors, and co-morbidities.

**Response:**

Thank you.

**Comment 1**

Additionally, the account for reverse causality in sensitivity analyses excluding individuals with CVD events in the first year and 2 years of follow up.

When individuals within the first 2 years of follow-up are excluded the event are substantially smaller. Which demonstrates a major limitation in this study of a relatively short follow up time. There appears to be on average only 2-3 years of follow up. I would suggest continue to emphasize this important limitation throughout the paper.

**Response:**

The updated dataset consists of 3,617 incident CVD cases with a median follow-up of more than five years. These new results support the same conclusions as those reported in our original manuscript but have allowed us to estimate associations much more precisely.

**Comment 2**

General Questions/Suggestions:

The additional analyses for reverse causality are helpful (supplement table 1). The number of cases is drastically reduced, so it can be difficult to draw many conclusions, however, it is interesting that the associations for quartile 4, the most active group, is attenuated in model 4. Do the authors believe this is all from reduced sample size and lower events? Could there be a potential that the curvilinear relationship (such as what is discussed by the authors with self-reported PA as the exposure) could exist with longer term follow-up? So in other words, is there potential that with longer follow-up we may see that leveling off/threshold at higher levels? Could only the more immediate risk of developing CVD (e.g. first 2 years of follow-up) to be linear with no leveling off of benefit? It would be interested to hear the authors' perspectives on this.

**Response:**

We now see no levelling off in the new analysis where we have 2,373 CVD events after removing the first two years of follow up.

**Comment 3**

Another area that would be useful for the authors to address is the quantification of moderate intensity activity and how high these estimates are for this sample. The authors try to address their methodology of how they quantified moderate and vigorous intensity and why they are higher, however, I think the justification could use stronger references. There are more specific comments below on this too. Because the authors use quartiles of total volume of PA, this concern would not influence the results and I believe what the authors did in using quartiles is good way to quantify groups for this study. Additionally, I think it is interesting and relevant to look at both total volume and moderate to vigorous intensity physical activity seperately. However, I would suggest for the authors to be cautious in language about the certainty of how much moderate intensity PA these participants are achieving, unless these values can be confirmed with further references or justifications. There are other cohorts that have objective measures of total MVPA (that includes MVPA accumulated at any bout length) that have much lower levels. The extreme differences appear difficult to justify that this all could be based on differences in device or the cut points used by other cohort studies. For example, supplement 1 of the recent meta analysis by Ekelund et al BMJ 2019 (<https://www.bmj.com/content/bmj/suppl/2019/08/19/bmj.l4570.DC1/ekeu048737.ww1.pdf>) provide a nice summary of several cohorts and their total MVPA min/day which ranged from 15 to about 37 min/day on average.

**Response:**

The accurate quantification of moderate intensity activity is an active and ongoing research challenge. We would first like to be clear that our estimates of MVPA time are from wrist-worn devices rather than hip-worn devices. The hip-worn devices in the Ekelund paper resulted in an average wear-time of just ~14 hours per day (in contrast to our median wear-time of 23.5 hours per day using wrist-worn devices). This means that the MVPA estimates from wrist-worn accelerometers will naturally be higher than estimates from hip-worn devices.

Even accounting for the difference between hip and wrist devices, we are confident that our estimates of MVPA time are plausibly comparable to previous literature. For example, the seminal Ekelund paper used a common, but stringent, ‘cut-point’ threshold to define MVPA time (1952 counts per minute - cpm). In contrast, a paper by Saint-Maurice, Troiano, Matthews, and colleagues used a less stringent threshold of 760 cpm [1]. The 760 cpm threshold is designed to capture a broader range of lifestyle and ambulatory activities and has been demonstrated to provide accurate estimates of time spent in MVPA [2-4]. Using the NHANHES cohort, the Ekelund paper reports a ~25 min/day estimate of MVPA time. However, if using the 760 cpm threshold, the estimate would instead be ~80 min/day of MVPA time. Our estimate of ~100 min/day of MVPA time, from wrist-worn devices, is therefore plausibly comparable to previous literature.

Our estimate of MVPA time is also comparable to other wrist-worn studies. For example, on a validation set of ~150 UK adults, we previously trained machine learned methods against a camera derived ground truth of moderate-intensity (>3MET) activities; and then deployed this to the UK Biobank population (Doherty, Nature Communications 2018)  and found a mean of ~75 min/day. In this work we decided to go with the more interpretable ‘cut-point’ approach for comparability with previous literature.

References used in rebuttal text:

[1] Saint-Maurice PF, Troiano RP, Matthews CE, Kraus WE. Moderate-to-Vigorous Physical Activity and All-Cause Mortality: Do Bouts Matter? J Am Heart Assoc. 2018;7(6).(pii):JAHA.117.007678. doi: 10.1161/JAHA.117..

[2] Matthew CE. Calibration of accelerometer output for adults. Med Sci Sports Exerc. 2005; 37:S512–S522.CrossrefMedlineGoogle Scholar

[3] Crouter SE, DellaValle DM, Haas JD, Frongillo EA, Bassett DR. Validity of ActiGraph 2‐regression model, Matthews cut‐points, and NHANES cut‐points for assessing free‐living physical activity. J Phys Act Health. 2013; 10:504–514.CrossrefMedlineGoogle Scholar

[4] Welk GJ, McClain JJ, Eisenmann JC, Wickel EE. Field validation of the MTI Actigraph and BodyMedia armband monitor using the IDEEA monitor. Obesity (Silver Spring). 2007; 15:918–928.CrossrefMedlineGoogle Scholar

**Changes to the paper:** We feel the above text would break the flow of the discussion text in this paper. However, we agree with the reviewer to be more cautious in this situation. We have therefore added the following sentence to the discussion section [Page 18, Lines 381-385]:

*In addition, the accurate quantification of levels of moderate PA from accelerometers remains an active challenge as we have had to rely on methods validated in relatively small validation studies [17,18]. There is a future need for large-scale validation studies in both healthy and diseased individuals [36]. In light of these considerations, we have pragmatically measured mean participant levels of moderate PA in this study.*

**Comment 4**

Introduction:

For the author's statement that total EE is conceivably the primary pathway through PA reduces risk of disease. Can they provide a better citation than #7 (Bassett et al.) This Bassett et al paper is great for the author's sentence before this on the measurement benefits of examining total PA, however this citation does not adequately justify the link between total EE/volume with disease risk. I would suggest justifying this statement with a stronger citation(s).

**Response:**

As suggested, we have cited the chapter, 6. Calories: Total Macronutrient Intake, Energy Expenditure, and Net Energy Stores from the book, Diet and Health: Implications for Reducing Chronic Disease Risk: Executive Summary, published by National Research Council (US) Committee on Diet and Health in 1989 has a detailed description about the link between total EE and risk for various diseases.

**Comment 5**

Methods:

Page 5: "We only included participants whose accelerometer data could be successfully calibrated, meaning that data recorded across participants and devices was comparable". It is unclear what this means. Does this indicate exclusion of individuals with extreme values and the high and low ends of their accelerometer data? And if so, can you further explain how you defined an outlier?

**Response:**

We agree that further clarification would be helpful here. We have therefore updated the text in the manuscript as follows [Page 7, Lines 161-166]:

*We only included participants whose accelerometer data could be successfully calibrated, meaning that different devices provide comparable data outputs [14]. We excluded participants with >1% clipped values, which occur when the sensor’s dynamic range of +-8g is exceeded before or after calibration [14]. In addition, we excluded participants with implausibly high activity values, defined as average vector magnitude scores >100mg.*

**Comment 6**

Assessment of physical activity:

I am surprised at how high the duration estimates of moderate intensity activity are for this sample. To demonstrate the validity of using the cut points of 100-400mg - are there other references that can help expand on this? I am not certain the citations provided are adequate. The #16 reference is a paper that includes validation of the ActiGraph and the GENEactiv accelerometers, not Axivity. The #17 reference is a paper that performed a validation of the Axivity among 20 healthy young adults (which is not the best representation of the current sample using in the UK biobank study) of 2 hours of standard behaviors that would not be very comprehensive for activities of daily living. Based on the evidence provided with these citations here, I have reservations on how well these cut points estimate time spent in moderate intensity PA.

**Response:**

As mentioned in response to comment 3, we are confident that our estimates of MVPA time are pragmatic. We have updated the discussion text as follows [Page 18, Lines 381-385]:

*In addition, the accurate quantification of levels of moderate PA from accelerometers remains an active challenge as we have had to rely on methods validated in relatively small validation studies [17,18]. There is a future need for large-scale validation studies in both healthy and diseased individuals [36]. In light of these considerations, we have pragmatically measured mean participant levels of moderate PA in this study.*

**Comment 7**

Statistical analysis looks good. On page 7 I would suggest adding the following wording to the first sentence for clarity as: "To address reverse causation we repeated the Cox regression analyses after removing [CVD events that occurred in] the first year and then two years of follow-up"

**Response:**

We have added the suggested text to the sentence.

**Comment 8**

Did the authors do any sensitivity analysis to check for differences between those excluded due to noncompliant accelerometer wear vs. those included? For example, were there higher rates of CVD in the compliant vs. non-compliant accelerometer groups? Any implications for those excluded due to non-compliance in this study?

**Response:**

We did not find higher rates of CVD in the compliant vs non-compliant accelerometer groups. We have added the following text to the Results section [Page 10, Lines 241-244]:

*Out of 6,614 participants without prevalent CVD who were excluded due to poor wear time, 262 (4.0%) were diagnosed with incident CVD during a median follow-up of 62.6 months (compared to 4.0% cases during a median follow-up of 61.9 months for the study participants).*

**Comment 9**

Results:

Page 8. Also able to estimate this rather quickly based on person years, for transparency and clarity, can the authors add the mean years±s.d. here and/or in the abstract?

**Response:**

We have added the median and interquartile range immediately after the person-years to the first paragraph of the Results section and the abstract too.

**Comment 10**

Page 8 table 1: the time spent in vigorous PA seems to match up much more closely with accelerometer-measured sum of both moderate AND vigorous PA from other cohort studies. Again, this lead back to my methods concern of how moderate PA was quantified as mentioned above.

**Response:**

As mentioned in response to comment 3, we are confident that our estimates of both moderate and vigorous PA time are pragmatic and plausibly comparable to previous literature.

**Comment 11**

Supplement Table 1:

Can the authors please add rows for the number of incident CVD and non-CVD in each quartile that would correspond to models 3 and 4? Knowing the events and total n's in each group would be useful.

**Response:**

We have now provided this information in Supplementary Table 1. However, when I added these numbers the table looked too complex to read. Therefore, we chose to include only the statistics for removal of first one and two years of follow-up because rest of the information was identical to that shown in Figure 1.

**Comment 12**

Below the table, it reads 'Model 4: Model 3 after removal of first two years…" should this actually be 'Model 4: Model 2 after removal of first two years…"?

**Response:**

The reviewer is correct. We have changed the text where relevant to reflect that**.**

Discussion:

**Comment 13**

Page 13, mid page – the “amounts/intensity” wording is slightly confusing here – can the authors simply use intensity? Or does the amount indicate something separately like dose of PA?

**Response:**

We originally used the term ‘amounts’ to indicate volume. Given that it is confusing we have now substituted this with the word ‘volume’.

**Comment 14**

Page 14: "using validated measures of activity type" What does type mean here? Do the authors mean intensity?

**Response:**

Yes, type here can be used to refer to intensity. We have replaced the term ‘type’ with ‘intensity’.

**Comment 15**

Page 14: "This could conceivably be explained by a different balance of the components of total PA across the different studies." Could the authors expand on this further for clarity?

**Response:**

To clarify this, we have added the following text after the sentence [Page 17, Lines 355-358]:

*In other words, if moderate and vigorous PA did have different effects on risk, then differences in observed associations of total PA with risk across studies could be explained by a disparity in the contribution of vigorous vs moderate PA to total PA across study samples.*

**Comment 16**

Page 15: The authors make good points about the differences of objective measures vs. self report. However, I would suggest removing or taming the last sentence in paragraph 1. "Our study is therefore likely to have more closely measured the activity that participants actually do, rather than what they can perceive what they do". The justifications/citations that the authors currently provide do not appear to adequately justify their estimates of moderate and vigorous activity for their study sample. Reference #27 that the authors provide here is focused on a single study of Actiheart and Bodymedia Armband. The Armband is no longer available and the Actiheart is not as widely used as other accelerometers for large cohort studies. It would be useful to have a few other citations to back up this point. Can the authors further justify/explain how moderate intensity was determined in the validation study? Please see my previous comments from the methods section.

**Response:**

While the Thompson study used devices that are now redundant, the underlying scientific finding from this study is still very much relevant when comparing device and self-reported measures of activity. However, we agree that providing another citation would be helpful. In response we have cited a paper from the Women’s Health Study where MVPA time was estimated as 512 mins/week using hip-worn devices versus 210 mins/week using self-reported measures.

As mentioned in previous responses, we are confident that moderate intensity activity has been measured in a justifiable and pragmatic manner. However, we agree that the aforementioned sentence could be better phrased. We have therefore changed the sentence as follows [Page 18, Lines 381-385]:

*In addition, the accurate quantification of levels of moderate PA from accelerometers remains an active challenge as we have had to rely on methods validated in relatively small validation studies [17,18]. There is a future need for large-scale validation studies in both healthy and diseased individuals [36]. In light of these considerations, we have pragmatically measured mean participant levels of moderate PA in this study.*

**Comment 17**

Can anything be said about the generalizability of the UK biobank as a strength or limitation? For instance, is it representative of SES, race/ethnic groups, and health status of the general population? The large sample size certainly helps with this.

**Response:**

We have added the following sentence to the ‘Strengths and Limitations’ section [Page 17, Lines 364-367]:

*Even though the characteristics of the UK Biobank participants may not be generalisable to other populations, it can be used to provide valid estimates of exposure-disease relationships due to its large sample size and multitude of exposures [29,30]*

**Comment 18**

It is also worth directly addressing that this study showed that those who do very high volumes of activity are not at an increased risk, and is a good contribution to the literature on this topic given the strength of the accelerometer measures to get objective total volume of activity.

**Response:**

We have added the following as the concluding sentence of paragraph 2 of the discussion section:

*Notably, we saw no evidence of a higher risk of CVD, particularly stroke, in those engaging in high levels of PA, including vigorous, in contrast to an observation of greater risk in such individuals in one large British cohort [25].*
