# Supplementary material for: Accelerometer measured physical activity and the incidence of cardiovascular disease: Evidence from the UK Biobank cohort study
Source: PLoS Med. 2021 Jan 12;18(1):e1003487. doi: 10.1371/journal.pmed.1003487 (PMC7802951; doi:10.1371/journal.pmed.1003487)
Supplement: Attachment — Submitted filename: Response to corrections requested.docx [file pmed.1003487.s009.docx]

1. You have stated that no prespecified plan exists for this study but also that "Analyses were planned once we submitted a request for data access and no data-driven analyses were included". We believe these statements are contradictory. If you had planned the analyses before undertaking this research please provide us with the protocol for these analyses and make the necessary edits to the manuscript.

**Response:** Since we did not have prespecified analysis plan we have revised the text in the manuscript as follows:

*This study did not have a prespecified analysis plan and no data-driven analyses were included.*

2. In the discussion you state "The UK Biobank cohort consists of mostly Caucasian participants living in less socioeconomically deprived areas who are likely to be more health-conscious than the general population". Please replace Caucasian with White. It is also misguided to assume that ethnicity and geolocation are related to health consciousness. I suggest you revise this to note that the Biobank is less ethnically diverse than the general UK population without further commenting on health consciousness.

**Response:**

As suggested, we have revised the above sentence as follows and hope this is okay:

*The UK Biobank cohort consists of mostly White participants living in less socioeconomically deprived areas.*

3. Please ensure that all Supporting Information files use the following format verbatim throughout the manuscript (including legends and all in-text citations): S1 Fig, S1 Table, S1 Text, S1 Data, etc. Each file type should start at 1 and increase numerically. Please note, however, that the file names themselves must use an underscore rather than a space, i.e. S1_Fig.tif, S1_Table.xlsx, S1_Text.doc, S1_Data.xlsx, S1_STROBE_Checklist.docx, etc.

**Response:** We have ensured that all supporting information files are in the format given above.
