# Supplementary figures and images for: Correction: Accelerometer measured physical activity and the incidence of cardiovascular disease: Evidence from the UK Biobank cohort study
Source: PLoS Med. 2021 Sep 29;18(9):e1003809. doi: 10.1371/journal.pmed.1003809 (PMC8480986; doi:10.1371/journal.pmed.1003809)

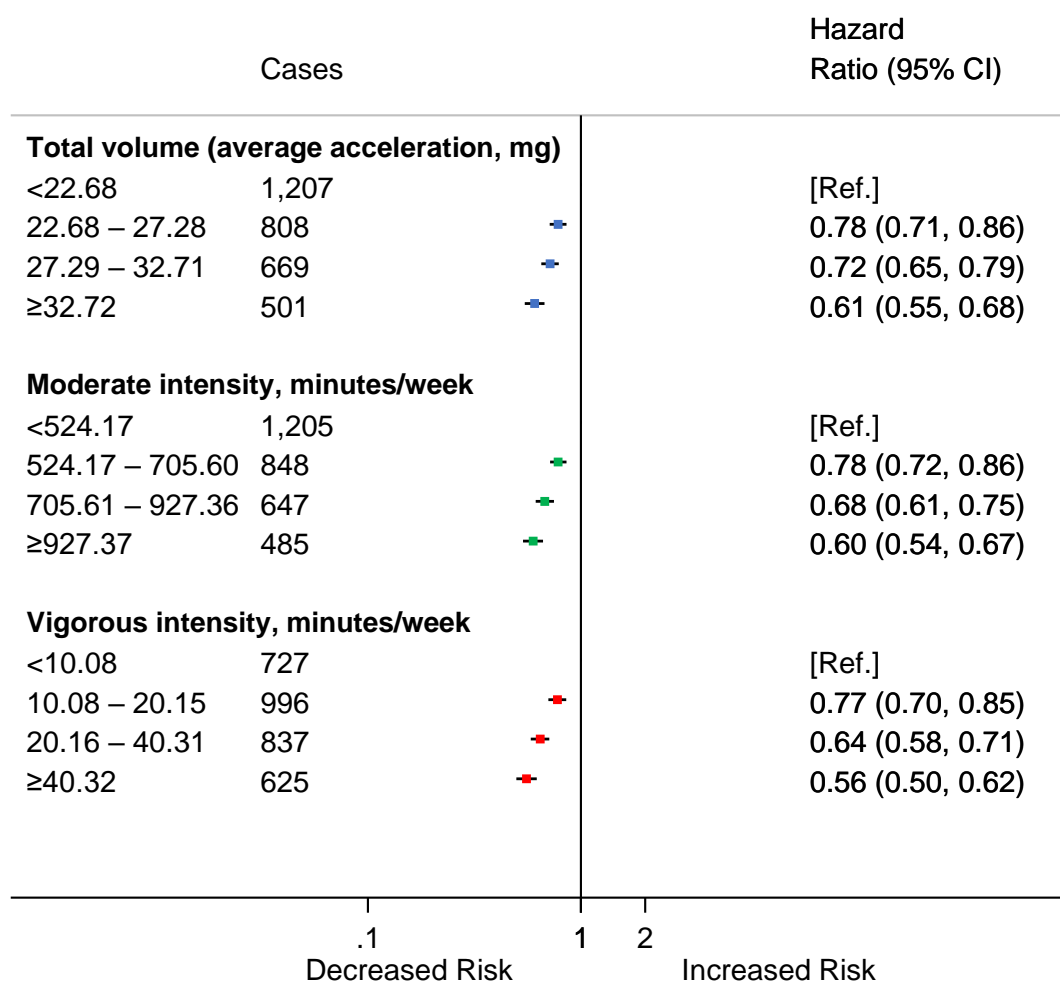

Supplement: S2 Fig — aAdjusted for age (stratified by 5-year age-at-risk intervals), sex, ethnicity, education, Townsend Deprivation Index, smoking, and alcohol consumption. bCancer (ICD codes: C01-C26, C30-C58, C60-C97, and D00-D48), diabetes mellitus (ICD codes: E10-E14), hypertension (ICD codes: I10), and chronic lower respiratory disease (ICD codes: J43 and J44.9). CVD, cardiovascular disease; HR, hazard ratio; ICD, International Classification of Diseases. https://doi.org/10.1371/journal.pmed.1003487.s007 (PDF) [file pmed.1003809.s001.pdf]
