# Supplementary material for: Correction: Accelerometer measured physical activity and the incidence of cardiovascular disease: Evidence from the UK Biobank cohort study
Source: PLoS Med. 2021 Sep 29;18(9):e1003809. doi: 10.1371/journal.pmed.1003809 (PMC8480986; doi:10.1371/journal.pmed.1003809)
Supplement: S1 Table — CVD, cardiovascular disease; HR, hazard ratio; PA, physical activity. (PDF) [file pmed.1003809.s002.pdf]

S1 Table. Adjusted hazard ratios for incident cardiovascular disease by quarters of average accelerometer-measured total volume (mg), moderate, and vigorous physical activity after removal of incident CVD occurring within one and two years of follow-up.

Total volume (mg)	<22.68	22.68 – 27.28	27.29-32.71	≥32.72
**Model 1**				
Incident CVD, n (%)	1,151 (5.2)	763 (3.4)	625 (2.8)	471 (2.1)
No CVD, n (%)	21,181 (94.9)	21,635 (96.6)	21,774 (97.3)	22,001 (97.9)
Hazard ratio (95% CI)	1.00 (Ref.)	0.78 (0.71, 0.86)	0.71 (0.65, 0.79)	0.61 (0.55, 0.69)
**Model 2**				
Incident CVD, n (%)	897 (4.1)	579 (2.6)	509 (2.3)	388 (1.7)
No CVD, n (%)	21,161 (95.9)	21,626 (97.4)	21,770 (97.8)	21,996 (98.3)
Hazard ratio (95% CI)	1.00 (Ref.)	0.76 (0.68, 0.84)	0.74 (0.67, 0.83)	0.65 (0.57, 0.73)
Moderate intensity physical activity (minutes/week)	<524.17	524.17-705.60	705.61-927.36	≥927.37
**Model 1**				
Incident CVD, n (%)	1,146 (5.3)	804 (3.5)	620 (2.7)	440 (2.0)
No CVD, n (%)	20,607 (94.7)	22,442 (96.5)	22,239 (97.3)	21,203 (98.0)
Hazard ratio (95% CI)	1.00 (Ref.)	0.79 (0.72, 0.86)	0.70 (0.63, 0.77)	0.58 (0.52, 0.65)
**Model 2**				
Incident CVD, n (%)	895 (4.2)	619 (2.7)	499 (2.2)	360 (1.7)
No CVD, n (%)	20,590 (95.8)	22,429 (97.3)	22,235 (97.8)	21,299 (98.3)
Hazard ratio (95% CI)	1.00 (Ref.)	0.78 (0.70, 0.86)	0.71 (0.64, 0.80)	0.61 (0.53, 0.69)
Vigorous intensity physical activity (minutes/week)	<10.08	10.08-20.15	20.16 -40.31	≥40.32
**Model 1**				
Incident CVD, n (%)	685 (5.3)	968 (3.9)	773 (2.9)	584 (2.3)
No CVD, n (%)	12,295 (94.7)	23,940 (96.1)	25,428 (97.1)	24,928 (97.7)
Hazard ratio (95% CI)	1.00 (Ref.)	0.81 (0.73, 0.89)	0.64 (0.58, 0.72)	0.57 (0.51, 0.65)
**Model 2**				
Incident CVD, n (%)	540 (4.2)	739 (3.0)	617 (2.4)	477 (1.9)
No CVD, n (%)	12,282 (95.8)	23,925 (97.0)	25,421 (97.6)	24,925 (98.1)
Hazard ratio (95% CI)	1.00 (Ref.)	0.78 (0.70, 0.87)	0.65 (0.58, 0.74)	0.60 (0.53, 0.68)

Note: Abbreviations: CVD =cardiovascular disease.

All models adjusted for age, sex, ethnicity, education, Townsend Deprivation Index, smoking, and alcohol consumption.

Model 1: After removal of CVD cases within first year of follow-up (Sample size: CVD= 3,010 and non-CVD=86,591)

Model 2: After removal of CVD cases within first two years of follow-up (Sample size: CVD=2,373 and non-CVD=88,926)
