# Supplementary material for: Correction: Accelerometer measured physical activity and the incidence of cardiovascular disease: Evidence from the UK Biobank cohort study
Source: PLoS Med. 2021 Sep 29;18(9):e1003809. doi: 10.1371/journal.pmed.1003809 (PMC8480986; doi:10.1371/journal.pmed.1003809)
Supplement: S2 Table — CVD, cardiovascular disease; HR, hazard ratio; PA, physical activity. (PDF) [file pmed.1003809.s003.pdf]

S2 Table. Hazard Ratios for the association between quarters of total volume of physical activity (mg) and incident cardiovascular disease with sequential adjustment for potential confounders and mediators

Adjustments	HR (95% CI)	HR (95% CI)	HR (95% CI)
**Milli-gravity (mg)**	**22.68 – 27.28 vs <22.68**	**27.29-32.71 vs <22.68**	**≥32.72 mg <22.68**
+ Age	0.74 (0.68, 0.80)	0.66 (0.60, 0.72)	0.55 (0.50, 0.61)
+ Sex	0.77 (0.70, 0.83)	0.70 (0.64, 0.76)	0.58 (0.52, 0.64)
+ Education	0.77 (0.71, 0.84)	0.70 (0.64, 0.77)	0.58 (0.52, 0.64)
+ Townsend Deprivation Index	0.78 (0.71, 0.85)	0.71 (0.65, 0.77)	0.58 (0.53, 0.64)
+ Ethnicity	0.78 (0.71, 0.84)	0.70 (0.64, 0.77)	0.58 (0.53, 0.64)
+ Smoking	0.78 (0.72, 0.85)	0.71 (0.65, 0.78)	0.59 (0.53, 0.65)
+ Alcohol consumption	0.79 (0.73, 0.86)	0.72 (0.66, 0.79)	0.60 (0.54, 0.66)
+ Hypertension	0.79 (0.73, 0.86)	0.73 (0.66, 0.80)	0.60 (0.54, 0.67)
+ Self rated health	0.84 (0.77, 0.91)	0.78 (0.71, 0.85)	0.66 (0.60, 0.73)
+ Body Mass Index	0.84 (0.79, 0.93)	0.81 (0.74, 0.89)	0.71 (0.64, 0.79)
+ Total cholesterol	0.87 (0.79, 0.95)	0.80 (0.73, 0.89)	0.71 (0.63, 0.79)
+ HDL cholesterol	0.86 (0.79, 0.94)	0.84 (0.76, 0.93)	0.73 (0.65, 0.82)
+ LDL cholesterol	0.86 (0.79, 0.94)	0.84 (0.76, 0.93)	0.73 (0.65, 0.82)
+ Triglycerides	0.86 (0.79, 0.94)	0.84 (0.76, 0.93)	0.73 (0.65, 0.82)
+ C-reactive protein	0.87 (0.79, 0.95)	0.85 (0.77, 0.94)	0.74 (0.66, 0.83)
+ HbA1c	0.87 (0.79, 0.96)	0.86 (0.76, 0.95)	0.74 (0.66, 0.83)
+ Red and processed meat intake	0.87 (0.79, 0.96)	0.86 (0.78, 0.95)	0.74 (0.66, 0.83)
+ Fresh fruit intake	0.87 (0.79, 0.96)	0.86 (0.78, 0.95)	0.74 (0.66, 0.84)
+ Cooked vegetable intake	0.87 (0.79, 0.96)	0.86 (0.78, 0.95)	0.74 (0.66, 0.84)

Abbreviations: HR, hazard ratio; CI, confidence interval; HbA1c, glycated haemoglobin
Note: C-reactive protein on log scale
