# Supplementary material for: Correction: Accelerometer measured physical activity and the incidence of cardiovascular disease: Evidence from the UK Biobank cohort study
Source: PLoS Med. 2021 Sep 29;18(9):e1003809. doi: 10.1371/journal.pmed.1003809 (PMC8480986; doi:10.1371/journal.pmed.1003809)
Supplement: S3 Table — CVD, cardiovascular disease; HR, hazard ratio; PA, physical activity. (PDF) [file pmed.1003809.s004.pdf]

S3 Table. Hazard Ratios for the association between quarters of moderate physical activity (minutes/week) and incident cardiovascular disease with sequential adjustment for potential confounders and mediators

**Adjustments**	**HR (95% CI)**	**HR (95% CI)**	**HR (95% CI)**
**Minutes/week**	**524.17-705.60 vs <524.17**	**705.61-927.36 vs <524.17**	**≥927.37 vs <524.17**
+ Age	0.75 (0.69, 0.81)	0.65 (0.59, 0.71)	0.53 (0.48, 0.59)
+ Sex	0.76 (0.70, 0.83)	0.67 (0.61, 0.73)	0.56 (0.51, 0.63)
+ Education	0.77 (0.71, 0.84)	0.67 (0.62, 0.74)	0.56 (0.51, 0.63)
+ Townsend Deprivation Index	0.77 (0.71, 0.84)	0.68 (0.62, 0.74)	0.57 (0.51, 0.63)
+ Ethnicity	0.77 (0.71, 0.84)	0.68 (0.62, 0.74)	0.57 (0.51, 0.63)
+ Smoking	0.78 (0.72, 0.85)	0.69 (0.63, 0.75)	0.57 (0.52, 0.64)
+ Alcohol consumption	0.79 (0.73, 0.86)	0.70 (0.64, 0.76)	0.58 (0.53, 0.65)
+ Hypertension	0.79 (0.73, 0.86)	0.70 (0.64, 0.77)	0.59 (0.53, 0.65)
+ Self rated health	0.83 (0.76, 0.90)	0.75 (0.68, 0.82)	0.63 (0.57, 0.70)
+ Body Mass Index	0.85 (0.78, 0.93)	0.78 (0.71, 0.86)	0.68 (0.61, 0.75)
+ Total cholesterol	0.85 (0.78, 0.92)	0.79 (0.72, 0.87)	0.67 (0.60, 0.75)
+ HDL cholesterol	0.85 (0.77, 0.93)	0.82 (0.74, 0.90)	0.68 (0.61, 0.77)
+ LDL cholesterol	0.85 (0.77, 0.93)	0.81 (0.74, 0.90)	0.68 (0.61, 0.77)
+ Triglycerides	0.85 (0.78, 0.94)	0.82 (0.75, 0.91)	0.70 (0.62, 0.78)
+ C-reactive protein	0.85 (0.77, 0.93)	0.84 (0.76, 0.93)	0.70 (0.62, 0.79)
+ HbA1c	0.85 (0.77, 0.93)	0.84 (0.76, 0.93)	0.70 (0.62, 0.79)
+ Red and processed meat intake	0.85 (0.77, 0.93)	0.84 (0.76, 0.93)	0.70 (0.62, 0.79)
+ Fresh fruit intake	0.85 (0.77, 0.93)	0.84 (0.76, 0.93)	0.70 (0.62, 0.79)
+ Cooked vegetable intake	0.85 (0.77, 0.93)	0.84 (0.76, 0.93)	0.70 (0.62, 0.79)

Abbreviations: HR, hazard ratio; CI, confidence interval; HbA1c, glycated haemoglobin
Note: C-reactive protein on log scale
